# Misophonia severity in university students: associations with negative emotionality, motor impulsivity, and checking

**DOI:** 10.1186/s40359-026-04390-z

**Published:** 2026-03-19

**Authors:** Murat Can Gumus, Efruz Pirdogan Aydin, Hasan Demirci, Omer Akil Ozer

**Affiliations:** 1 Department of Psychiatry, Akdagmadeni Sehit Sinan Babacan State Hospital, Yozgat, Türkiye; 2 Department of Psychiatry, Sisli Hamidiye Etfal Training and Research Hospital, University of Health Sciences, Huzur Mah. Cumhuriyet ve Demokrasi Cad. No:1 Sarıyer, Istanbul, Türkiye; 3 Department of Psychology, University of Health Sciences, Istanbul, Türkiye

**Keywords:** Misophonia, Prevalence, Path analysis, Indirect effect, Impulsivity, Checking

## Abstract

**Background:**

Misophonia is characterized by strong negative emotional and behavioral reactions to specific everyday sounds and is associated with significant functional impairment. Previous studies have primarily focused on associations between misophonia and negative affect (e.g., depression, anxiety, and stress), whereas less is known about how misophonia severity relates to impulsivity and specific obsessive–compulsive symptom dimensions in non-clinical populations.

**Objective:**

The present study aimed to estimate the prevalence of Misophonia Questionnaire (MQ) cut-off–defined elevated misophonia symptoms among university students, describe emotional and behavioral responses to trigger sounds, and examine whether motor impulsivity and checking are involved in the associations between depression, anxiety, stress symptoms, and misophonia severity.

**Methods:**

A total of 562 university students aged 18–30 years participated in this cross-sectional study. Participants completed the MQ, Depression Anxiety Stress Scales (DASS-21), Barratt Impulsiveness Scale–Short Form (BIS-11-SF), and Maudsley Obsessional–Compulsive Inventory (MOCI). Model-based path analyses were conducted to examine direct and indirect associations between negative affect symptoms and misophonia severity through motor impulsivity and checking.

**Results:**

Sixteen point 4% of participants met the MQ cut-off for elevated misophonia symptoms. Misophonia severity showed positive associations with depression, anxiety, stress symptoms, motor impulsivity, and checking. Path analyses indicated that anxiety and stress symptoms were directly associated with misophonia severity, while depressive symptoms were associated only indirectly. Significant indirect associations were observed through motor impulsivity and checking, although the indirect pathway via checking was not significant for anxiety.

**Conclusions:**

Misophonia severity is associated not only with negative affect but also with impulsivity- and compulsivity-related dimensions. These findings highlight the relevance of motor impulsivity and checking as behavioral dimensions linked to misophonia severity and underscore the importance of considering multidimensional psychopathological features when assessing misophonia in non-clinical populations.

**Supplementary Information:**

The online version contains supplementary material available at 10.1186/s40359-026-04390-z.

## Introduction

The word *misophonia* is derived from the Greek words *miso* (hatred) and *phonia* (sound) [1]. According to the recent Delphi consensus, misophonia is characterized by disproportionate emotional reactions to triggering sounds, accompanied by urges to avoid or prevent these sounds, which can impair daily functioning [2]. Misophonia symptoms are reported to typically emerge in childhood or adolescence [3, 4] and rarely in adulthood [5]. In studies conducted with university students, the rate of clinically significant misophonia ranges from 6% to 20% [6–8], while in general-population or nationally representative samples, this rate varies between 4.6% and 12.8% [9–11]. Consistent with this variability, a recent systematic review reported that prevalence estimates range widely across countries and studies (approximately 5%–34.7%), likely due to differences in sampling, assessment instruments, and cut-off criteria [12]. Despite increasing recognition of misophonia as a distinct clinical phenomenon, a universally accepted definition and standardized assessment are still lacking.

Misophonia is commonly triggered by specific everyday sounds, typically rhythmic and repetitive human-generated sounds—such as lip-smacking, chewing, coughing, yawning, snoring, whistling—or mechanical stimuli such as clinking cutlery, crumpling paper, ticking clocks, or keyboard sounds [2, 4, 13, 14]. In individuals with misophonia, core affective responses such as intense anger and disgust—and, at times, co-occurring anxiety—may arise in reaction to these sounds, often accompanied by autonomic symptoms including palpitations, sweating, respiratory discomfort, flushing, or trembling [15].

Beyond these immediate affective and physiological reactions, individuals with misophonia often experience heightened hypervigilance and anticipatory anxiety related to the unpredictability of trigger sounds, which can contribute to significant distress and functional impairment [4, 16, 17]. To manage this distress, many individuals engage in avoidance or escape behaviors, particularly in shared or social environments, which are associated with disruptions in family, social, and academic functioning [18]. In addition to avoidance, individuals may adopt other coping strategies—such as warning others, mimicking trigger sounds, or using headphones or background noise—although these strategies may provide only temporary relief [19].

Moreover, mixed-methods research suggests that the misophonic experience is often characterized by appraisals of boundary violation and loss of autonomy, accompanied by defensive anger and disgust, often alongside impulsive action tendencies and safety behaviors [20]. These findings underscore the role of cognitive appraisals in shaping responses during trigger exposure, ranging from internalizing interpretations (e.g., self-blame or attempts to suppress distress) to externalizing beliefs such as blaming others or assuming intentionality behind the sound-producing behavior [21–23]. Accordingly, at higher levels of distress, some individuals may display a tendency toward impulsive anger responses, hostility, and verbal aggression [13, 14, 24], with the intensity and expression of these reactions varying across interpersonal and contextual factors [25]. Although overt physical aggression (e.g., hitting others or objects) appears to be relatively uncommon [14], some individuals report verbal outbursts [24], as well as aggressive thoughts or mental imagery during exposure to trigger sounds [26].

Misophonia, first described in the field of audiology, has only recently been discussed within the psychiatric literature [1]. Consequently, its nosological status remains unclear, including whether misophonia constitutes an independent psychiatric disorder or reflects a symptom pattern arising within other psychopathologies. Although this issue has not been fully resolved, recent consensus work increasingly suggests that misophonia may represent a distinct diagnostic entity [2]. At the same time, misophonia shows considerable symptom-level overlap with anxiety disorders, mood disorders, post-traumatic stress disorder (PTSD), obsessive–compulsive disorder (OCD), and attention-deficit/hyperactivity disorder (ADHD), and such diagnoses are frequently reported among individuals with misophonia [4, 27–29].

Beyond its clinical phenomenology, growing evidence suggests that misophonia is a condition with neurological, psychological, and audiological components. Neuroimaging findings reinforce this conceptualization: misophonia is not limited to auditory hypersensitivity but involves increased activation and connectivity in regions responsible for emotional and cognitive processing, including the limbic system, the default mode network (DMN), and the anterior cingulate cortex (ACC). In particular, heightened activity in the anterior insula, amygdala, and orbitofrontal cortex (OFC) has been associated with difficulties regulating emotional responses, increased excitability, and compulsive–impulsive tendencies [30–33].

More broadly, misophonia has been increasingly linked to elevated negative affect, including depression, anxiety, and stress symptoms. Stress- and anxiety-related mechanisms are of particular relevance to misophonia, given the prominence of hypervigilance, physiological arousal, and fight-or-flight tendencies during trigger exposure [2, 34]. Empirical evidence further indicates that anxiety shows a positive association with misophonia severity across multiple studies [7, 35–37].

Moreover, dimensional anxiety-related constructs such as anxiety sensitivity have been implicated in misophonia severity through obsessive–compulsive symptoms [36], and may also moderate the association between misophonia and aggressive behavioral responses [24]. Similarly, in youth samples, transdiagnostic risk factors such as distress intolerance and anxiety sensitivity have been associated with greater misophonia severity, and improvements in distress intolerance may represent a mechanism of symptom change during transdiagnostic cognitive-behavioral treatment [38]. Recent findings suggest that the severity of misophonia is more closely related to perceived stress than to traumatic stress symptoms, that it is not related to the number of negative life events, and is particularly associated with a state of hyperarousal/hypervigilance [34].

In contrast, the relationship between depressive symptoms and misophonia severity appears less consistent across studies. Although several studies have reported positive associations between depression severity and misophonia severity [39, 40], other findings indicate non-significant relationships [41]. Rather than exerting a direct effect on symptom severity, depressive symptoms may be linked to misophonia through chronic stress–related processes [37]. Notably, depression has been shown to relate more strongly to functional impairment associated with misophonia than to misophonia severity itself [23]. Taken together, these findings suggest that depression, anxiety, and stress may exhibit distinct and partially independent patterns of association with misophonia.

Misophonic reactions have been discussed above as potentially involving anger outbursts and sudden behavioral consequences. Such aggressive and impulsive responses are among the most distressing aspects of the condition for affected individuals [13, 22], and may also have important implications for treatment approaches [42, 43].

Supporting this view, a recent neuropsychological study reported elevated behavioral impulsivity and higher anxiety levels in individuals with misophonia compared with controls [41], suggesting that impulsivity may be more relevant to how individuals respond to trigger sounds than to the number of sounds perceived as triggering. Along similar lines, impulse-control difficulties have been shown to fully mediate the relationship between neuroticism and misophonia severity [44], and impulsivity has been proposed as a key contributor to anger outbursts in misophonia [23]. Moreover, defensive anger in misophonia appears to be accompanied by autonomic nervous system activation and reactive impulsive behaviors [20].

Although emerging evidence suggests that impulsivity dimensions beyond attentional impulsivity may be relevant to misophonia, this area remains insufficiently explored [37]. Taken together, these findings highlight impulsivity as a central dimension in misophonia. Accordingly, the present study focused on motor impulsivity as an index of action-oriented, rapid reactive responding during trigger exposure.

Another important dimension associated with misophonia involves obsessive–compulsive symptoms. Previous studies have shown that misophonia may co-occur with OCD and obsessive–compulsive personality traits, with such comorbidities reported at meaningful rates [4, 14, 28, 45]. Beyond comorbid diagnostic overlap, misophonia has also been conceptualized as a condition in which exposure to aversive stimuli is associated with intense stress, leading some individuals to engage in obsessive–compulsive–like behaviors as maladaptive coping strategies [14, 46, 47]. However, although obsessive–compulsive symptoms are associated with misophonia, findings regarding the specific symptom dimensions involved remain inconsistent across studies [36, 39, 40, 48].

In this context, checking, one of the core and most frequently observed symptoms of OCD, is theoretically linked to fundamental processes such as reducing uncertainty, seeking reassurance, and preventing perceived harm [49]. The presence of checking behaviors across anxiety and depressive disorders further suggests that this behavior may play a transdiagnostic role in the regulation of emotional distress [50, 51]. Accordingly, checking may be conceptualized as a behavioral manifestation of attempts to manage uncertainty and perceived threat, potentially offering a pathway through which emotional distress contributes to greater misophonia severity. From this perspective, checking may represent a relevant pathway statistically linking negative emotional states to misophonia severity.

Overall, misophonia has been associated with heightened negative affect and behavioral reactivity, with impulsivity- and compulsivity-related processes potentially contributing to variability in symptom expression. However, it remains unclear to what extent these processes explain the associations between misophonia severity and symptoms of depression, anxiety, and stress. Therefore, the present study had two main aims: (i) to estimate the prevalence of MQ cut-off–defined elevated misophonia symptoms among university students and to characterize common trigger sounds as well as the emotional and behavioral responses to these triggers, and (ii) to test a theoretically driven parallel mediation model in which motor impulsivity and checking were examined as mediators of the associations between depression, anxiety, and stress symptoms and misophonia severity.

Based on the literature, we hypothesized that: (1) anxiety and stress symptoms would be positively associated with misophonia severity, whereas associations with depressive symptoms would be comparatively weaker or more variable; (2) higher levels of depression, anxiety, and stress symptoms would be associated with higher motor impulsivity and checking; (3) motor impulsivity and checking would each be positively associated with misophonia severity; and (4) motor impulsivity and checking would show significant indirect effects (i.e., parallel mediation) in the associations between depression, anxiety, and stress symptoms and misophonia severity.

## Materials and methods

### Design

In this study, a cross-sectional and correlational design was employed. A path analytic model was tested within the SEM framework to examine the mediating roles of impulsivity and compulsivity in the relationship between misophonia severity and negative emotionality (i.e., depression, anxiety, and stress). Additionally, descriptive analyses were conducted to present the distribution and severity of misophonia symptoms in the sample.

### Sample

Of the students participating in the study (*n* = 562), 63.3% were female (*n* = 356), 36.7% were male (*n* = 206), and their average age was 19.77 ± 1.46 years. The inclusion criteria for the study were being between the ages of 18 and 30 and agreeing to participate. Of the students included in the study, 36.7% were from the psychology department (*n* = 206), 26.7% were from dentistry (*n* = 150), 22.4% were from medicine (*n* = 126), and 14.2% were from nursing (*n* = 80). Exclusion criteria for the study were: (i) being outside the 18–30 age range; (ii) using a hearing aid or reporting hearing impairment; (iii) having a diagnosis of autism spectrum disorder (pervasive developmental disorder, autistic disorder, asperger syndrome, etc.) or ADHD. During data collection, 23 participants were excluded at baseline due to incomplete questionnaires or missing data. A total of 579 volunteers were initially recruited. Seventeen participants were excluded due to age outside the inclusion range (*n* = 9), a self-reported ADHD diagnosis (*n* = 4), or hearing loss (*n* = 4), resulting in a final sample of 562 participants.

### Assessments

#### Sociodemographic data form

It is a form consisting of 14 questions, prepared by the researchers for the study, to determine the sociodemographic characteristics of the participants, which the participants filled out. It consists of questions about the age, gender, education level, marital status, smoking, alcohol, substance use, psychiatric and physical disease history of the individuals, hearing status/whether they have ever applied to an otolaryngologist due to hearing loss, tinnitus, family history of misophonia/psychiatric disease.

### Maudsley obsessional–compulsive inventory (MOCI)

The MOCI is a self-report measure assessing obsessive–compulsive symptoms [52]. The original scale includes subscales covering checking, cleaning, slowness, and doubt. The Turkish adaptation was conducted by Erol and Savaşır (1988), who expanded the instrument by adding a rumination dimension and additional items, resulting in a 37-item version. Items are answered in a dichotomous format (True/False), with higher total scores indicating greater obsessive–compulsive symptom severity. The Turkish version has demonstrated good reliability (Cronbach’s α = 0.86), with subscale α values ranging from 0.61 to 0.84 [53]. In the present sample, Cronbach’s α was 0.841 for the total scale. The subscale α coefficients were 0.655 for checking, 0.611 for cleaning, 0.471 for slowness, 0.457 for doubt, and 0.775 for rumination.

### Depression anxiety stress scale (DASS-21)

The DASS-21 is the 21-item short form of the Depression Anxiety Stress Scales, and its psychometric properties have been supported in previous studies [54]. It assesses depression, anxiety, and stress levels over the past week using three 7-item subscales scored on a 4-point Likert scale (0–3), with each subscale ranging from 0 to 21. In the Turkish validation study, Cronbach’s α internal consistency coefficients were reported as 0.87 for the depression subscale, 0.85 for the anxiety subscale, and 0.81 for the stress subscale [55]. In the present sample, Cronbach’s α was 0.866 for the total scale, and the subscale α coefficients were 0.854 for depression, 0.816 for anxiety, and 0.793 for stress.

### Barratt impulsivity scale–short form (BIS-11-SF)

The Barratt Impulsivity Scale is a widely used self-report measure of impulsivity in both clinical and non-clinical populations, originally developed by Barratt (1959) [56], and later revised as the BIS-11 by Patton et al. (1995) [57]. The BIS-11 includes three subscales: attentional impulsivity, motor impulsivity, and non-planning impulsivity. The Turkish validity and reliability study of the BIS-11 was conducted by Güleç et al. (2008). Based on the BIS-11, a 15-item short form (BIS-11-SF) was developed by selecting the five highest-loading items from each subscale. In the original short-form study, internal consistency was reported as Cronbach’s α = 0.82 for the total scale, with subscale α values ranging from 0.64 to 0.80. Items are rated on a 4-point Likert scale, and higher scores indicate greater impulsivity [58]. In the present sample, Cronbach’s α was 0.674 for the total scale, and the subscale α coefficients were 0.769 for attentional impulsivity, 0.570 for motor impulsivity, and 0.783 for non-planning impulsivity.

### Misophonia questionnaire (MQ)

The MQ is a self-report measure developed by Wu et al. (2014) to assess misophonia-related symptoms and emotional/behavioral responses to triggering sounds in both clinical and community samples [7]. The scale includes a 7-item Misophonia Symptom subscale and a 10-item Misophonia Emotions and Behaviors subscale, rated on a 5-point Likert scale (0–4), yielding a total score ranging from 0 to 68, with higher scores indicating greater misophonia symptom severity. The final section, the Misophonia Severity Scale, is rated from 1 to 15 and provides an overall severity index; this score is not included in the MQ total score. According to Wu et al. (2014), scores ≥ 7 on this severity rating indicate at least moderate sound sensitivity associated with significant interference and have been used as a cut-off for clinically significant misophonia symptoms. In the Turkish validity and reliability study conducted by Sakarya and Çakmak (2021), the MQ demonstrated a three-factor structure, including misophonia symptoms, emotions/behaviors–avoidance and internalization, and emotions/behaviors–aggression and externalization. The authors reported good internal consistency for the total scale, with a Cronbach’s α of 0.89 and subscale α values of 0.79, 0.85, and 0.83, respectively [59]. In the present sample, Cronbach’s α was 0.783 for the total scale, and the subscale α coefficients were 0.803 for misophonia symptoms, 0.851 for emotions/behaviors–avoidance and internalization, and 0.839 for emotions/behaviors–aggression and externalization. For the purpose of group comparisons, participants scoring ≥ 7 on the Misophonia Severity Scale were classified as having cut-off–defined elevated misophonia symptoms.

### Procedure

Firstly, approval was obtained from the Ethics Committee of the University of Health Sciences, Hamidiye Scientific Research Ethics Committee (Decision Number: 2023-23/336). Data were collected between 10.10.2023 and 15.12.2023. The study was conducted by recruiting students from the Faculty of Medicine, Faculty of Dentistry, Faculty of Nursing, and Faculty of Psychology at University of Health Sciences, using the convenience sampling method. Care was taken to select courses with high student participation in these faculties. After the course owner was informed about the study, it was planned that the researcher would administer the survey and scales to the students in the classroom environment at the beginning or end of the course. After the necessary information was provided, the Informed Consent Form and study scales were distributed to the prospective participants. The volunteer participants read and approved the Consent Form. The participants were informed that they should not include personal information on the forms and scales that would reveal their identity. After the study forms were collected, they were numbered, and the data were transferred to the researcher’s electronic environment.

### Statistical analysis

For all statistical analyses, SPSS Statistics for Windows, Version 26.0 (IBM Corp., Armonk, NY, USA) and AMOS Version 22.0 (IBM Corp.) programs were used. In descriptive statistics, mean and standard deviation values for continuous variables, as well as frequencies and percentages for categorical variables, are reported. In order to evaluate the differences between two groups (those with and without elevated misophonia symptoms), chi-square test (χ²) was applied for categorical variables, independent samples t-test for continuous variables when they showed a normal distribution, and Mann–Whitney U test for continuous variables when they did not show a normal distribution. To evaluate the relationships between misophonia severity and clinical variables, Pearson correlation analysis was employed for data exhibiting normal distribution. In contrast, Spearman correlation analysis was used for data that did not show normal distribution.

Path analysis within a SEM framework was conducted using AMOS to examine the direct and indirect effects of depression, anxiety, and stress on misophonia severity, with all variables specified as observed (scale/subscale) scores. DASS-21 Depression, Anxiety, and Stress subscales were entered as independent variables, MQ total score as the dependent variable, and motor impulsivity (BIS-11-SF motor impulsivity) and checking (MOCI-checking) as mediators. Depression, anxiety, and stress were allowed to covary in the model.

Depression, anxiety, and stress were retained as separate predictors to preserve their differential clinical meaning. Mediator selection for the final model was guided by both theoretical considerations and the empirical pattern observed at the subscale level. Motor impulsivity was selected as the impulsivity indicator because, among the BIS-11-SF dimensions, it showed the strongest association with misophonia severity and was also considered theoretically relevant to behavioral reactivity in misophonia. Among the MOCI dimensions, several subscales were associated with misophonia severity, including checking and rumination. Checking was retained in the final model because it was considered theoretically more consistent with uncertainty reduction and threat regulation processes, whereas alternative SEM models showed poorer fit.

Model parameters were estimated using maximum likelihood estimate (MLE). Univariate normality was acceptable (skewness and kurtosis between − 1 and + 1), and multivariate normality was evaluated using Mardia’s coefficient (CR < 10). To account for minor deviations from multivariate normality, indirect effects were tested using bias-corrected bootstrap estimation with 5,000 resamples and 95% confidence intervals. Multicollinearity among depression, anxiety, and stress was assessed using variance inflation factors (VIFs), which ranged from 2.04 to 2.53. Single-mediator models were tested first, followed by the final parallel mediation model including both mediators simultaneously. Model fit was evaluated using χ²/df (CMIN/df), Comparative Fit Index (CFI), Tucker–Lewis Index (TLI), Normed Fit Index (NFI), and Root Mean Square Error of Approximation (RMSEA). Statistical significance was set at *p* < 0.05.

## Results

### Samples

Among the students who participated in the study, 92 individuals (16.4%) were identified as having elevated misophonia symptoms. Participants were divided into two groups: those with elevated misophonia symptoms (*n* = 92) and those without elevated misophonia symptoms (*n* = 470), and were compared in terms of sociodemographic data and clinical scales. The groups did not show any significant differences in terms of gender (χ² = 0.776, *p* = 0.378) and age (t = 0.283, *p* = 0.777). When the participants were evaluated in terms of the people they lived with, it was observed that the majority of them lived in dormitories (48.7%) or with their families (41.5%) (χ² = 0.059, *p* = 0.808). There was no significant difference between the two groups in terms of variables such as employment status, smoking, and alcohol use, presence of physical illness, and personal or family history of psychiatric illness (*p* > 0.05). However, the family history of misophonia was found to be significantly higher in participants with misophonia (χ² = 11.915, *p* = 0.001) (Table 1).

**Table 1:** Sociodemographic characteristics of the total sample and comparisons between groups

Variables	Total Sample (*n* = 562)	MQ ≥ 7 (*n* = 92)	MQ < 7 (*n* = 470)	Test Statistic	*p*
Sex, Female, *n* (%)	356 (63.3)	62 (67.4)	294 (62.6)	0.776	0.378
Age, M ± SD	19.77 ± 1.46	19.80 ± 1.45	19.76 ± 1.47	0.283	0.777
Living with, *n* (%)					
Alone	11 (2.0)	1 (1.1)	10 (2.1)		
Family	233 (41.5)	39 (42.4)	194 (41.3)		
Roommate	43 (7.6)	5 (5.4)	38 (8.1)		
Dormitory	274 (48.7)	47 (51.1)	227 (48.3)	0.059	0.808
Other	1(0.2)	0	1(0.2)		
Working, Yes, *n* (%)	40 (7.1)	6 (6.5)	34 (7.2)		
Smoking, Yes, *n* (%)	96 (17.1)	16 (17.4)	80 (17.0)	0.007	0.931
Alcohol Usage, Yes, *n* (%)	93 (16.5)	13 (14.1)	80 (17.0)	0.466	0.495
Physical Disease, Yes, *n* (%)	19 (3.4)	2 (2.2)	17 (3.6)	—	0.374
Psychiatric disease, Yes, *n* (%)	52 (9.3)	7 (7.6)	45 (9.6)	0.354	0.552
Family History of Psychiatric Disease, *n* (%)	57(10.1)	8 (8.7)	49 (10.4)	0.253	0.615
Family History of Misophonia, *n* (%)	55(9.8)	18 (19.6)	37 (7.9)	11.915	0.001

### Frequency of misophonic trigger sounds

The most commonly reported trigger sounds among participants with elevated misophonia symptoms, based on the combined “often” and “always” responses, were eating sounds (79.3%), throat sounds such as throat clearing (60.9%), nasal sounds (58.6%), and repetitive tapping sounds (52.2%). Environmental sounds were also commonly reported (42.4%), whereas speech sounds were less common (15.2%). These findings indicate that eating and mouth-throat sounds were particularly prominent in the elevated misophonia symptoms group (Supplemental Table S1).

### Frequency of misophonic reactions

Based on the combined “often” and “always” responses, the most frequently reported reactions among participants with elevated misophonia symptoms were feeling annoyed (91.3%), feeling angry (72.8%), leaving the disturbing environment (63.0%), and avoidance behavior (63.0%). Anxiety/distress was also common (52.2%), while covering the ears was reported by 43.5% of participants. Violent thoughts were reported by 39.2% of participants. Although physical aggression was relatively uncommon (13.0%), verbal aggression was more frequent (46.7%). (Supplemental Table S2).

### Comparisons of clinical scales

The misophonia group exhibited significantly higher MQ symptoms, emotion-behavior subscales, and total scores compared to the control group (*p* < 0.001). Significant increases were also observed in the MOCI subscales in the misophonia group. The most significant differences were observed in the doubt (*p* < 0.001), rumination (*p* = 0.006), and checking (*p* = 0.007) subscales. The MOCI total score was also higher in the misophonia group (18.93 ± 7.08 vs. 15.94 ± 7.64; *p* < 0.001). Depression, anxiety, and stress levels were also significantly higher in the misophonia group (*p* = 0.001). While motor impulsivity (*p* = 0.001) and lack of planning (*p* = 0.018) were found to be significantly higher in the misophonia group, no significant difference was found between the groups in terms of attentional impulsivity and BIS-11 total score (*p* = 0.159 and *p* = 0.425, respectively) (Supplemental Table S3).

### Correlation with misophonia severity and clinical scales

Misophonia severity showed moderate positive correlations with checking (*r* = 0.306, *p* < 0.01), slowness (*r* = 0.326, *p* < 0.01), rumination (*r* = 0.347, *p* < 0.01), and MOCI total score (*r* = 0.387, *p* < 0.01). Weak positive correlations were found with cleanliness (*r* = 0.231, *p* < 0.01) and doubt (*r* = 0.265, *p* < 0.01) subscales. Misophonia severity also showed moderate positive correlations with depression (*r* = 0.327, *p* < 0.01), anxiety (*r* = 0.432, *p* < 0.01), stress (*r* = 0.475, *p* < 0.01), and DASS-21 total score (*r* = 0.461, *p* < 0.01). In addition, misophonia severity was moderately positively correlated with motor impulsivity (*r* = 0.315, *p* < 0.01) and weakly positively correlated with attention impulsivity (*r* = 0.220, *p* < 0.01). However, no significant correlation was found between misophonia severity and non-planning impulsivity (*p* > 0.05). (Supplemental Table S4).

### Path analysis

**Fig. 1:**
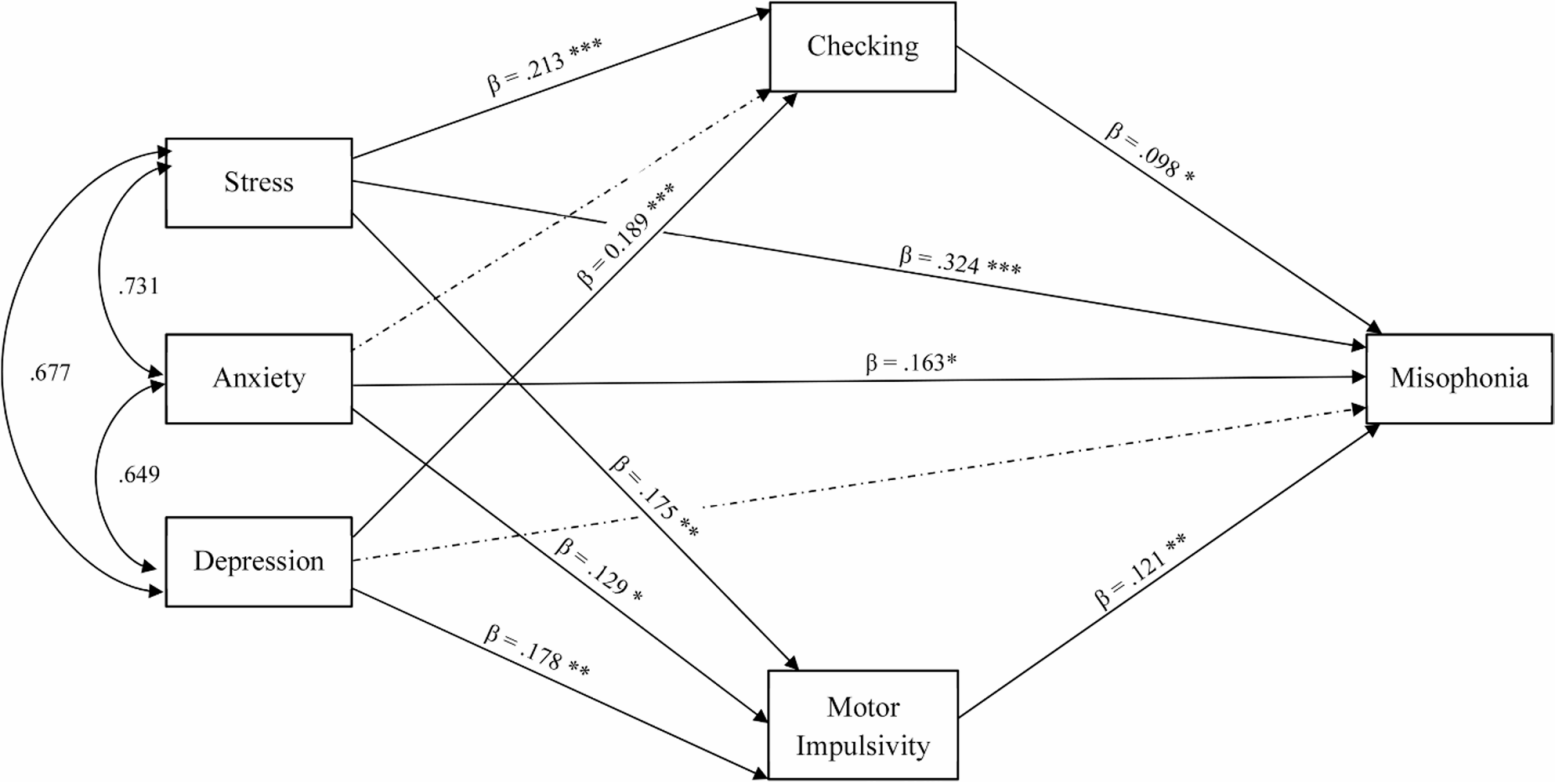
Structural equation modelling showing standardized coefficient. Dash line is non-significant parameter. *** *p*<0.001, ** 0.001-0.01, *<0.05

Figure 1 presents the final SEM with standardized coefficients. The final model demonstrated an acceptable to excellent fit to the data (χ²/df = 3.696, CFI = 0.998, TLI = 0.967, NFI = 0.997, RMSEA = 0.069).

Table 2 presents the MLEs of the structural paths in the final model. All three negative affective variables were significantly associated with motor impulsivity (depression: β = 0.178, *p* = 0.001; anxiety: β = 0.129, *p* = 0.028; stress: β = 0.175, *p* = 0.004). Similarly, depression (β = 0.189, *p* < 0.001) and stress (β = 0.213, *p* < 0.001) were positively associated with checking, whereas the association between anxiety and checking did not reach statistical significance (β = 0.109, *p* = 0.059). Both mediators were significantly related to misophonia severity. Checking (β = 0.098, *p* = 0.017) and motor impulsivity (β = 0.121, *p* = 0.003) were independently associated with misophonia severity.

**Table 2:** Structural path coefficients estimated using maximum likelihood estimation (MLE)

Path	MLE				
	B	SE(B)	CR	β	*p*
Depression → Motor impulsivity	0.101	0.031	3.270	0.178	0.001
Anxiety → Motor impulsivity	0.080	0.036	2.202	0.129	0.028
Stress → Motor impulsivity	0.106	0.037	2.877	0.175	0.004
Depression → Checking	0.079	0.022	3.531	0.189	< 0.001
Anxiety → Checking	0.050	0.027	1.887	0.109	0.059
Stress → Checking	0.096	0.027	3.560	0.213	< 0.001
Checking → Misophonia	0.635	0.265	2.397	0.098	0.017
Motor Impulsivity → Misophonia	0.580	0.193	3.009	0.121	0.003
Anxiety → Misophonia	0.486	0.168	2.889	0.163	0.004
Stress → Misophonia Depression→ Misophonia	0.949 -0.235	0.172 0.144	5.509 -1.637	0.324 − 0.087	< 0.001 0.102

Model Fit indices were as follows: CMIN/df = 3.696, CFI = 0.998, TLI = 0.967, NFI = 0.997, RMSEA = 0.069

*Abbreviation*: *B* Unstandardized estimate, *MLE* Maximum Likelihood Estimate, *SE* Standard error, *CR* Critical ratio, *β* Standardized estimate

Bootstrap analyses indicated significant indirect effects of depression, anxiety, and stress on misophonia severity (Table 3). Regarding direct effects, anxiety (β = 0.163, *p* = 0.009) and stress (β = 0.324, *p* < 0.001) showed significant direct associations with misophonia severity. The direct path from depression to misophonia severity was non-significant (β = -0.087, *p* = 0.079).

**Table 3:** Direct, indirect, and total effects of depression, anxiety, and stress on misophonia severity

Predictor → MQ	Effect	β	Boot SE	BC 95% CI [L, U]	Boot BC *p*
Depression	Total	− 0.047	0.051	[-0.151, 0.048]	0.340
	Direct	− 0.087	0.050	[-0.186-0.009]	0.079
	Indirect	0.040	0.015	[0.016, 0.076]	< 0.001
Anxiety	Total	0.190	0.058	[0.070, 0.302]	0.001
	Direct	0.163	0.059	[0.043, 0.277]	0.009
	Indirect	0.026	0.013	[0.007, 0.059]	0.005
Stress	Total	0.366	0.056	[0.254, 0.483]	< 0.001
	Direct	0.324	0.059	[0.211, 0.443]	< 0.001
	Indirect	0.042	0.016	[0.016, 0.081]	< 0.001

*Abbreviation*: *BC 95% CI* Bias-corrected Bootstrap Confidence Interval, *MQ* Misophonia Questionnaire, *SE* Standart Error, *β* Standardized estimate. Indirect effects were tested using bootstrapping with 5,000 resamples and BC 95% confidence intervals. Indirect effects were considered statistically significant when the BC 95% CI did not include zero

Bootstrap analyses indicated significant indirect effects of depression on misophonia severity through both motor impulsivity (β = 0.061, 95% BC CI [0.012, 0.144]) and checking (β = 0.050, 95% BC CI [0.010, 0.122]). Stress also exerted significant indirect effects via motor impulsivity (β = 0.062, 95% BC CI [0.013, 0.147]) and checking (β = 0.061, 95% BC CI [0.012, 0.144]). Anxiety showed a significant indirect effect through motor impulsivity (β = 0.047, 95% BC CI [0.006, 0.125]), whereas the indirect path via checking was marginal and did not reach statistical significance (β = 0.032, 95% BC CI [0.000, 0.095]) (Table 4).

**Table 4:** Specific indirect effects of depression, anxiety, and stress on misophonia severity via motor impulsivity and checking

Indirect Effect	β	Boot SE	BC 95% CI [L, U]	Boot BC *p*
Depression → Motor Impulsivity → MQ	0.061	0.030	[0.012, 0.144]	0.011
Depression → Checking → MQ	0.050	0.027	[0.010, 0.122]	0.012
Anxiety → Motor Impulsivity → MQ	0.047	0.029	[0.006, 0.125]	0.023
Anxiety → Checking → MQ	0.032	0.022	[0.000, 0.095]	0.047
Stress → Motor Impulsivity → MQ	0.062	0.033	[0.013, 0.147]	0.007
Stress → Checking → MQ	0.061	0.033	[0.012, 0.144]	0.011

*BC 95% CI* Bias-corrected Bootstrap Confidence Interval, *MQ* Misophonia Questionnaire, *SE* Standard Error, *β* Standardized estimate

Indirect effects were tested using bootstrapping with 5,000 resamples and BC 95% confidence intervals

## Discussion

This study examined the prevalence of MQ cut-off–defined elevated misophonia symptoms in university students, characterized common triggers and reactions, and tested a parallel mediation model linking depression, anxiety, and stress symptoms to misophonia severity via motor impulsivity and checking. Taken together, these findings provide a comprehensive overview of both the phenomenological features of misophonia and the emotional and behavioral correlates of symptom severity.

In the present study, the prevalence of MQ cut-off–defined elevated misophonia symptoms was 16.4% (*n* = 92) based on the MQ severity cut-off of ≥ 7, a criterion previously used in university student samples and associated with similar prevalence rates in the literature [7, 8, 60]. However, prevalence estimates of misophonia vary substantially depending on the operational definitions and cut-off criteria employed. Several studies indicate that although misophonia symptoms are relatively common, clinically significant functional impairment is observed only in a subset of cases [8, 9, 12, 61].

The determination of what constitutes “clinical” misophonia therefore remains controversial. Previous research has shown that severity-based cut-offs alone may overestimate clinically meaningful cases, whereas incorporating emotional, behavioral, and functional impairment criteria yields substantially lower and more clinically relevant estimates [9, 62, 63]. This emphasis on functional impact is consistent with DSM and ICD diagnostic frameworks, which require significant social, academic, or occupational impairment for diagnosis.

When trigger sounds in misophonia are examined, it has been observed that triggers originating from the mouth and nose (e.g., mouth smacking, eating, sniffing, breathing, throat clearing) are at the forefront [9, 13, 64, 65]. In addition, mechanical and rhythmic sounds, such as clock sounds, keyboard sounds, and the sound of a pencil clicking, have also been reported as frequent triggers. In our study, 79.3% of the participants reported “often” or “always ” responses to eating sounds, 60.9% to throat sounds, and 58.6% to nasal sounds. These findings are consistent with the existing literature. One possible reason why these mouth and nose sounds stand out as more prominent triggers is that they are both commonly encountered in daily life and generally occur in the context of social relationships, especially in environments where people in the immediate vicinity are present [25, 66]. In other words, such sounds carry not only auditory but also relational and contextual meanings.

After encountering triggering sounds, people may experience some emotional or behavioral reactions. Irritability, discomfort, anxiety, disgust, and anger are the most common emotions in misophonia [20, 25, 35, 67]. Schröder and colleagues reported that getting angry was the most common misophonic reaction among participants, with 59.9% experiencing verbally aggressive behaviors, 28.6% damaging objects, and 12% engaging in physical aggression [14]. In a recent study by Larsen et al., the most common reactions in the Norwegian version of the MQ were getting angry (83.2%) and leaving the environment (56.3%). However, feeling sad and depressed (16.3%) and physical aggression (3.5%) were reported as the least experienced reactions [68]. In our study, based on the combined “often” and “always” response categories, 91.3% of individuals with elevated misophonia symptoms reported feeling annoyed, 72.8% reported feeling angry, and 52.2% reported anxiety/distress in response to trigger sounds. Sadness/depressed mood was the least commonly reported emotional response (31.5%). In addition, 13.0% of individuals with elevated misophonia symptoms reported being physically aggressive, whereas 46.7% reported being verbally aggressive. It is known that anxiety sensitivity plays a leading role in anger expression and aggressive reactions in misophonic individuals [24, 69]. At the same time, aggressive reactions observed in misophonic individuals may negatively affect the clinical course. Aggression has been associated with a lack of response to treatment, deterioration in occupational and interpersonal functioning, and additional psychiatric disorders [3, 70]. Anger control difficulties and aggressive behaviors in misophonic individuals stand out as a priority area for treatment; however, there are not yet enough studies on this subject [42]. In one of the few studies in this field, it was reported that anger intensity decreased after cognitive behavioral therapy applied to misophonic female university students [43].

Our study contributes to the literature by examining distinct psychopathological mechanisms linking negative affective symptoms to misophonia severity through motor impulsivity and checking. SEM indicated that anxiety was associated with misophonia severity both directly and indirectly via motor impulsivity, whereas stress exerted both direct effects and indirect effects mediated by motor impulsivity and checking. In contrast, no direct effect of depression on misophonia severity was observed; instead, its influence emerged only through mediator variables. These findings suggest that different dimensions of negative affect may be linked to misophonia through partially distinct pathways.

Previous research has reported inconsistent findings regarding the relationship between depression and misophonia. While some studies have identified significant associations between depressive symptom severity and misophonia [7, 25, 37, 40], others have failed to demonstrate a direct relationship [71]. The current findings are consistent with the possibility that depressive symptoms may be indirectly, rather than directly via heightened arousal, related to misophonia severity.

Misophonia is typically characterized by heightened physiological arousal, irritability, and avoidance responses to trigger sounds, reflecting increased sympathetic activation [34]. In contrast, depression is commonly associated with psychomotor slowing, withdrawal, low energy, and reduced responsiveness to environmental stimuli. Given these divergent neurophysiological and behavioral profiles, the absence of a direct association between depression and misophonia severity in the present study appears theoretically consistent.

Instead, depressive symptomatology may influence misophonia through cognitive–emotional processes such as rumination and cognitive rigidity, which are prominent features of depression [72–74]. These processes may be involved in the appraisal and emotional salience of trigger sounds, thereby indirectly exacerbating misophonic responses when combined with impulsive or compulsive tendencies.

Consistent with prior research, anxiety emerged as the most robust affective correlate of misophonia severity. A growing body of research indicates that misophonia is particularly linked to anxiety, with higher anxiety levels associated with greater symptom severity [35, 75] and heightened emotional reactivity [16]. Quek et al. found significant relationships between misophonia severity and depression, anxiety, and stress levels measured using the DASS-21 scale; however, multiple regression analyses indicated that only anxiety significantly predicted misophonia severity [71]. Phenomenologically, misophonia shares key features with anxiety and phobic disorders, including anticipatory tension and heightened physiological reactivity [76].

Importantly, the present findings indicate that the indirect effects of anxiety and stress on misophonia severity operate through partially distinct mechanisms. Anxiety was indirectly associated with misophonia severity via motor impulsivity but not through checking, whereas stress exerted indirect effects through both pathways. The anxiety dimension assessed in this study primarily captures bodily autonomic symptoms (e.g., tremor, palpitations, dry mouth) and is consistent with evidence that impulsivity in anxiety disorders stems primarily from increased negative emotion rather than cognitive worry [77]. Accordingly, anxiety-related autonomic arousal may preferentially facilitate rapid, action-oriented responses. Supporting this interpretation, anxiety has been identified as the strongest predictor of overall impulsivity in young and middle-aged individuals [78], and recent network-based studies have highlighted motor impulsivity as a central transdiagnostic node linking anxiety and depressive symptoms [79].

By comparison, stress may represent a more sustained affective state, allowing for both impulsive reactions and the emergence of maladaptive regulatory strategies, including checking. In line with this interpretation, recent findings suggest that perceived stress is closely associated with misophonia severity, supporting the view that stress appears to be a salient phenotypic feature of misophonia [34]. Collectively, these findings underscore the presence of differentiated behavioral pathways through which anxiety, stress, and depression contribute to misophonia severity.

The misophonia scale used in the current study includes two basic emotion–behavior subscales: aggression–externalization and avoidance–internalization. While items related to avoidance show conceptual overlap with anxiety and obsessive-compulsive symptoms, items reflecting physical and verbal aggression are more closely aligned with motor impulsivity. A recent study reported that individuals diagnosed with misophonia had higher anxiety and stress levels as well as elevated behavioral impulsivity scores, and that comorbid OCD and ADHD diagnoses were more common than in the control group [41]. These findings were interpreted as suggesting that misophonia may involve both impulsivity- and compulsivity-related features, potentially reflecting psychological and neural processes that overlap with those implicated in ADHD and OCD.

In our study, checking showed a significant mediating effect on misophonia severity. This finding suggests that misophonia may be closely related to cognitive control processes involved in managing uncertainty and perceived threat. At the neurobiological level, previous research has suggested overlapping brain circuits between misophonia and obsessive–compulsive symptoms, particularly involving the OFC and ACC [30, 31, 33].

Within this framework, triggering sounds may become especially intolerable for individuals with heightened checking tendencies, as these stimuli represent uncontrollable external inputs that conflict with an increased need for certainty and mental control. When such conflicts persist, individuals may increasingly rely on avoidance and suppression strategies at both cognitive and behavioral levels, thereby contributing to the maintenance and exacerbation of the misophonic cycle. Indeed, Eijsker et al. reported that patients with misophonia did not exhibit a generalized impairment in response inhibition tasks; instead, they showed a response bias favoring accuracy over speed in a stop-signal task [80]. This pattern reflects a cognitive bias associated with perfectionistic and compulsive tendencies and further strengthens the link between misophonia and checking. Moreover, sustained checking may undermine confidence in memory and perceived cognitive control, thereby increasing uncertainty and distress [49]. Similarly, Daniels et al. (2020) showed that exposure to misophonic triggers is associated with reduced cognitive control performance and heightened distress [16].

According to SEM results, the impulsivity sub-dimension most strongly associated with misophonia severity was motor impulsivity. This finding suggests that sudden, uncontrolled, and externally directed reactions, which are central features of misophonia, play a prominent role in behavioral impulsivity. In particular, reactions such as restlessness, irritability, anger, and aggression to triggering sounds overlap with the sudden behaviors measured by motor impulsivity, consistent with recent findings highlighting defensive anger and impulsive action tendencies in misophonia [20]. This suggests that misophonic reactions are primarily directed toward sudden, external, and emotionally charged stimuli and may be related to heightened sensitivity to external sensory–perceptual inputs rather than to deficits in internal cognitive control. Indeed, neuropsychological evidence suggests that impairments in response inhibition and attention in misophonia emerge primarily during emotional trigger exposure. In contrast, these functions are relatively preserved in neutral situations [16, 76, 80, 81].

Although several related dimensions were associated with misophonia severity, the final focus on checking and motor impulsivity was guided primarily by theoretical considerations and further supported by the empirical pattern observed in the present data. Accordingly, the final model should be interpreted as one theoretically and empirically supported configuration, rather than as evidence that other related dimensions are not relevant.

This study has some limitations. The study employed a cross-sectional, epidemiological design, and participants were assessed solely through self-report measures. First, because the study was cross-sectional, causal inferences cannot be made. Accordingly, the identified indirect pathways should be interpreted as statistical rather than causal or temporal relationships. Second, the use of self-report measures may have introduced response bias and may have affected the accuracy of symptom classification. In addition, the misophonia scale used in this study assessed only auditory triggers and did not include visual or multisensory triggers. No audiological tests were administered to evaluate hearing function; therefore, co-occurring auditory problems such as hyperacusis or tinnitus may have gone undetected. Furthermore, impulsivity and compulsivity were assessed only through self-report scales and could not be objectively evaluated using cognitive tests. Finally, misophonia status was determined based on the cut-off score of the scale used, and the lack of standardized diagnostic criteria in the literature may have influenced the number of participants classified as having elevated misophonia symptoms. These findings provide conceptually and clinically important directions for future research. However, the limitations of the study should be carefully considered when interpreting the results.

In conclusion, the prevalence of MQ cut-off–defined elevated misophonia symptoms among university students was 16.4%, indicating that such symptoms were relatively common in this population. Path analyses indicated that misophonia severity was directly associated with anxiety and stress levels and indirectly associated with these affective dimensions through checking and motor impulsivity. In contrast, depressive symptoms were not directly associated with misophonia severity but were indirectly related through mediating psychopathological processes. Taken together, these findings suggest that impulsive and compulsive tendencies may represent relevant behavioral dimensions associated with misophonia severity. From a clinical perspective, the results highlight the potential relevance of considering impulsivity and checking behaviors alongside negative affective symptoms in the conceptualization of misophonia. These findings may also inform future intervention research targeting emotion regulation, impulse control, and maladaptive checking strategies, particularly in light of the indirect pathways identified between negative affect and misophonia severity.

## Supplementary Information

Supplementary Material 1.

## Data Availability

The datasets generated and/or analyzed during the current study are not publicly available due to ethical/privacy restrictions but are available from the corresponding author on reasonable request.

## Abbreviations

ACC: Anterior Cingulate Cortex
ADHD: Attention-Deficit/Hyperactivity Disorder
BIS-11-SF: Barratt Impulsivity Scale-Short Form
DASS-21: Depression, Anxiety, and Stress Scale
DMN: Default Mode Network
MOCI: Maudsley Obsessional Compulsive Inventory
MQ: Misophonia Questionnaire
OFC: Orbito-Frontal Cortex
OCD: Obsessive–Compulsive Disorder
PTSD: Post-Traumatic Stress Disorder
SEM: Structural Equation Modeling
