# Supplementary material for: Misophonia severity in university students: associations with negative emotionality, motor impulsivity, and checking
Source: BMC Psychol. 2026 Mar 19;14:618. doi: 10.1186/s40359-026-04390-z (PMC13123145; doi:10.1186/s40359-026-04390-z)
Supplement: Supplementary file 1 — Supplementary Material 1. [file 40359_2026_4390_MOESM1_ESM.docx]

**Supplemental Table S1**

*Frequencies of Misophonic Sound Triggers in the Elevated Misophonia Symptoms Group (MQ ≥ 7)*

| Triggers | Never | | Rarely | | Sometimes | | Often | | Always | |
| --- | --- | --- | --- | --- | --- | --- | --- | --- | --- | --- |
|  | *n* | % | *n* | % | *n* | % | *n* | % | *n* | % |
| People Eating | 1 | 1.1 | 9 | 9.8 | 9 | 9.8 | 23 | 25 | 50 | 54.3 |
| Repetitive Tapping | 5 | 5.4 | 12 | 13 | 27 | 29.4 | 18 | 19.6 | 30 | 32.6 |
| Rustling | 12 | 13 | 19 | 20.7 | 29 | 31.5 | 13 | 14.1 | 19 | 20.7 |
| Nasal Sounds | 9 | 9.8 | 11 | 12 | 18 | 19.6 | 25 | 27.1 | 29 | 31.5 |
| Throat Sounds | 4 | 4.3 | 19 | 20.7 | 13 | 14.1 | 19 | 20.7 | 37 | 40.2 |
| Consonants and Vowels | 45 | 48.9 | 22 | 23.9 | 11 | 12 | 6 | 6.5 | 8 | 8.7 |
| Environmental Sounds | 13 | 14.1 | 21 | 22.8 | 19 | 20.7 | 14 | 15.2 | 25 | 27.2 |

**Supplemental Table S2**

*Frequencies of Misophonic Reactions in Elevated Misophonia Symptoms Group (MQ ≥ 7)*

| Misophonic Reactions | Never | | Rarely | | Sometimes | | Often | | Always | |
| --- | --- | --- | --- | --- | --- | --- | --- | --- | --- | --- |
|  | *n* | % | *n* | % | *n* | % | *n* | % | *n* | % |
| Leave Environment | 4 | 4.4 | 9 | 9.8 | 21 | 22.8 | 27 | 29.3 | 31 | 33.7 |
| Avoid | 5 | 5.5 | 8 | 8.7 | 21 | 22.8 | 20 | 21.7 | 38 | 41.3 |
| Cover Ears | 20 | 21.7 | 16 | 17.4 | 16 | 17.4 | 17 | 18.5 | 23 | 25.0 |
| Anxious/Distressed | 9 | 9.8 | 15 | 16.3 | 20 | 21.7 | 23 | 25.0 | 25 | 27.2 |
| Sad/Derpessed | 22 | 23.9 | 27 | 29.6 | 14 | 15.2 | 15 | 16.3 | 14 | 15.2 |
| Annoyed | 0 | 0.0 | 2 | 2.2 | 6 | 6.5 | 21 | 22.8 | 63 | 68.5 |
| Violent Thoughts | 21 | 22.8 | 22 | 23.9 | 13 | 14.1 | 18 | 19.6 | 18 | 19.6 |
| Angry | 4 | 4.3 | 10 | 10.9 | 11 | 12.0 | 27 | 29.3 | 40 | 43.5 |
| Psychically Aggresive | 49 | 53.3 | 20 | 21.7 | 11 | 12.0 | 8 | 8.7 | 4 | 4.3 |
| Verbally Agressive | 7 | 7.6 | 16 | 17.4 | 16 | 17.4 | 22 | 23.9 | 21 | 22.8 |

**Supplemental Table S3**

*Comparisons of Clinical Scale Scores Between Groups*

| Variable | With Misophonia (MQ≥7, n = 92) | Without Misophonia | Test value | p |
| --- | --- | --- | --- | --- |
| MQ-Symptom | 16.55 ± 5.75 | 11.21 ± 5.60 | 7.534 | <.001 |
| MQ Emotions and Behaviors Scale-1 | 15.41 ± 5.18 | 8.88 ± 5.26 | 9.332 | <.001 |
| MQ Emotions and Behaviors Scale-2 | 8.12 ± 3.99 | 4.49 ± 3.67 | 7.68 | <.001 |
| MQ Total | 40.09 ± 11.23 | 24.58 ± 12.14 | 9.757 | <.001 |
| MOCI-Checking | 3.50 ± 1.93 | 2.98 ± 2.06 | 2.695 | .007 |
| MOCI-Cleaning | 4.25 ± 2.18 | 3.72 ± 2.11 | 2.201 | .028 |
| MOCI-Slowness | 2.58 ± 1.51 | 2.18 ± 1.57 | 2.473 | .013 |
| MOCI-Doubting | 3.80 ± 1.53 | 3.06 ± 1.67 | 3.95 | <.001 |
| MOCI-Rumination | 4.80 ± 2.49 | 4.00 ± 2.50 | 2.752 | .006 |
| MOCI-Total | 18.93 ± 7.08 | 15.94 ± 7.64 | 3.616 | <.001 |
| Depression | 8.28 ± 5.24 | 6.39 ± 4.76 | 3.303 | .001 |
| Anxiety | 7.77 ± 4.66 | 5.50 ± 4.33 | 4.445 | <.001 |
| Stress | 9.53 ± 4.51 | 7.03 ± 4.44 | 4.898 | <.001 |
| DASS-21-Total | 25.59 ± 12.54 | 18.92 ± 12.02 | 4.855 | <.001 |
| Non-planning | 9.74 ± 2.85 | 10.54 ± 2.80 | 2.361 | .018 |
| Motor Impulsivity | 10.50 ± 2.67 | 9.50 ± 2.76 | 3.447 | .001 |
| Attentional Impulsivity | 9.40 ± 2.64 | 9.10 ± 2.79 | 1.408 | .159 |
| BIS-11-Total | 29.64 ± 6.57 | 29.14 ± 6.49 | 0.798 | .425 |

*Note. Abbreviation= BIS-11: Barratt Impulsiveness Scale; DASS-21: Depression Anxiety Stress Scale-21; MOCI: Maudsley Obsessional-Compulsive Inventory; MQ: Misophonia Questionnaire; MQ Emotions and Behaviors Scale-1: Avoidance and Internalization; MQ Emotions and Behaviors Scale-2: Aggression and Externalization.*

**Supplemental Table S4**

*Correlations Among Clinical Scales in the Total Sample*

| Variable | 1 | 2 | 3 | 4 | 5 | 6 | 7 | 8 | 9 | 10 | 11 | 12 | 13 | 14 | 15 | 16 | 17 | 18 |
| --- | --- | --- | --- | --- | --- | --- | --- | --- | --- | --- | --- | --- | --- | --- | --- | --- | --- | --- |
| 1. MQ-Sym | — |  |  |  |  |  |  |  |  |  |  |  |  |  |  |  |  |  |
| 2. MQ-EB1 | 0.604** | — |  |  |  |  |  |  |  |  |  |  |  |  |  |  |  |  |
| 3. MQ-EB2 | 0.480** | 0.625** | — |  |  |  |  |  |  |  |  |  |  |  |  |  |  |  |
| 4. MQ Total | 0.847** | 0.887** | 0.775** | — |  |  |  |  |  |  |  |  |  |  |  |  |  |  |
| 5. MOCI-Chk | 0.273** | 0.273** | 0.239** | 0.306** | — |  |  |  |  |  |  |  |  |  |  |  |  |  |
| 6. MOCI-Cln | 0.222** | 0.209** | 0.168** | 0.231** | 0.332** | — |  |  |  |  |  |  |  |  |  |  |  |  |
| 7. MOCI-Slw | 0.267** | 0.294** | 0.260** | 0.326** | 0.621** | 0.400** | — |  |  |  |  |  |  |  |  |  |  |  |
| 8. MOCI-Dbt | 0.214** | 0.239** | 0.224** | 0.265** | 0.553** | 0.298** | 0.473** | — |  |  |  |  |  |  |  |  |  |  |
| 9. MOCI-Rum | 0.277** | 0.337** | 0.280** | 0.347** | 0.627** | 0.329** | 0.650** | 0.515** | — |  |  |  |  |  |  |  |  |  |
| 10. MOCI-Tot | 0.328** | 0.358** | 0.302** | 0.387** | 0.814** | 0.613** | 0.806** | 0.718** | 0.844** | — |  |  |  |  |  |  |  |  |
| 11. Depression | 0.220** | 0.318** | 0.315** | 0.327** | 0.420** | 0.217** | 0.435** | 0.347** | 0.550** | 0.529** | — |  |  |  |  |  |  |  |
| 12. Anxiety | 0.347** | 0.398** | 0.373** | 0.432** | 0.386** | 0.298** | 0.449** | 0.376** | 0.580** | 0.554** | 0.648** | — |  |  |  |  |  |  |
| 13. Stress | 0.363** | 0.417** | 0.444** | 0.475** | 0.425** | 0.286** | 0.459** | 0.403** | 0.591** | 0.572** | 0.673** | 0.727** | — |  |  |  |  |  |
| 14. DASS-21 Tot | 0.345** | 0.423** | 0.427** | 0.461** | 0.462** | 0.294** | 0.504** | 0.420** | 0.643** | 0.618** | 0.874** | 0.877** | 0.903** | — |  |  |  |  |
| 15. BIS-NP | -0.070 | 0.147** | 0.228** | -0.046 | -0.045 | -.170** | -0.015 | -0.112** | 0.071 | -0.062 | 0.129** | 0.089* | 0.002 | 0.081 | — |  |  |  |
| 16. BIS-Mot | 0.238** | 0.286** | 0.267** | 0.315** | 0.262** | 0.207** | 0.382** | 0.219** | 0.393** | 0.384** | 0.391** | 0.391** | 0.391** | 0.440** | 0.219** | — |  |  |
| 17. BIS-Att | 0.138** | 0.214** | 0.217** | 0.220** | 0.187** | 0.057 | 0.234** | 0.122** | 0.328** | 0.251** | 0.360** | 0.313** | 0.286** | 0.356** | 0.495** | 0.516** | — |  |
| 18. BIS-Tot | 0.114** | 0.171** | 0.216** | 0.190** | 0.170** | 0.035 | 0.257** | 0.099* | 0.329** | 0.239** | 0.363** | 0.324** | 0.271** | 0.359** | 0.731** | 0.729** | 0.846** | — |

*Note. MQ = Misophonia Questionnaire; MQ-Sym = MQ Symptom Scale; MQ-EB1 = MQ Emotions and Behaviors Scale-1; MQ-EB2 = MQ Emotions and Behaviors Scale-2. MOCI = Maudsley Obsessive–Compulsive Inventory; Chk = Checking; Cln = Cleaning; Slw = Slowness; Dbt = Doubting; Rum = Rumination; Tot = Total. Dep/Anx/Str = Depression/Anxiety/Stress subscales of DASS-21; DASS-Tot = DASS-21 Total Score. BIS = Barratt Impulsiveness Scale-11; BIS-NP = Non-planning; BIS-Mot = Motor Impulsivity; BIS-Att = Attentional Impulsivity; BIS-Tot = Total.*

**p<0.05, ** p<0.01*
